# Current-induced Néel order switching facilitated by magnetic phase transition

**DOI:** 10.1038/s41467-022-29170-2

**Published:** 2022-03-28

**Authors:** Hao Wu, Hantao Zhang, Baomin Wang, Felix Groß, Chao-Yao Yang, Gengfei Li, Chenyang Guo, Haoran He, Kin Wong, Di Wu, Xiufeng Han, Chih-Huang Lai, Joachim Gräfe, Ran Cheng, Kang L. Wang

**Affiliations:** 1 Department of Electrical and Computer Engineering, University of California, Los Angeles, CA 90095 USA; 2 Songshan Lake Materials Laboratory, Dongguan, Guangdong 523808 China; 3 Department of Electrical and Computer Engineering, University of California, Riverside, CA 92521 USA; 4 School of Physical Science and Technology, Ningbo University, Ningbo, 315211 China; 5 CAS Key Laboratory of Magnetic Materials and Devices, Ningbo Institute of Materials Technology and Engineering, Chinese Academy of Sciences, Ningbo, 315201 China; 6 Max Planck Institute for Intelligent Systems, Heisenbergstraße 3, Stuttgart, 70569 Germany; 7 Department of Materials Science and Engineering, National Tsing Hua University, Hsinchu, 30013 Taiwan; 8 Beijing National Laboratory for Condensed Matter Physics, Institute of Physics, Chinese Academy of Sciences, Beijing, 100190 China

**Keywords:** Spintronics, Magnetic devices, Electronic and spintronic devices

## Abstract

Terahertz (THz) spin dynamics and vanishing stray field make antiferromagnetic (AFM) materials the most promising candidate for the next-generation magnetic memory technology with revolutionary storage density and writing speed. However, owing to the extremely large exchange energy barriers, energy-efficient manipulation has been a fundamental challenge in AFM systems. Here, we report an electrical writing of antiferromagnetic orders through a record-low current density on the order of 10^6^ A cm^−2^ facilitated by the unique AFM-ferromagnetic (FM) phase transition in FeRh. By introducing a transient FM state via current-induced Joule heating, the spin-orbit torque can switch the AFM order parameter by 90° with a reduced writing current density similar to ordinary FM materials. This mechanism is further verified by measuring the temperature and magnetic bias field dependences, where the X-ray magnetic linear dichroism (XMLD) results confirm the AFM switching besides the electrical transport measurement. Our findings demonstrate the exciting possibility of writing operations in AFM-based devices with a lower current density, opening a new pathway towards pure AFM memory applications.

## Abstract (web summary)

Electrical manipulation of antiferromagnetic order is crucial for future memory devices, but existing switching schemes require a large current density. Here, the authors achieve record low current density switching in FeRh by taking advantage of its antiferromagnetic to ferromagnetic phase transition.

## Introduction

Antiferromagnetic materials are promising candidates for the high-density and fast-speed spintronic devices in the future^1–4^, the analogy to current ferromagnet (FM)-based spintronic applications such as hard disk drive and magnetic random-access memory^5,6^. The zero-stray field of antiferromagnets (AFMs) makes them to be robust against the magnetic interaction between different unit cells, which can further help to scale down the size of spintronic devices towards sub-nanometers; and the THz-range magnetic resonance frequency of AFMs (exchange mode) inspires the revolution of the speed for spintronic devices from nanoseconds to picoseconds timescale^7–11^.

Electrical manipulation of the antiferromagnetic order (Néel vector) is crucial for the high-density memory devices^12–20^. However, the energy-efficient electrical switching of the Néel vector is still a major challenge. Different from the FM, AFMs with zero magnetization are not sensitive to the external field and the electrical current. Fundamentally, for the fully-compensated AFM spin sublattices, the damping-like torque can only cant the AFM sublattices and then forms a small net magnetic moment, where the field-like torque plays a major role for the Néel vector switching^21^. Once the AFM sublattices are canted away from the −180^o^ alignment, an additional exchange torque arises from the AFM exchange interaction between sublattices, which cancels the field-like torque and thus reduces the efficiency of current-driven Néel vector switching. Usually, an ultrahigh current density of 10^8^ A cm^−2^ is required for the Néel vector switching, either by the current-induced staggered field^12^ or spin–orbit torque^13^. Moreover, the electrical transport measurement of Néel vector switching with such high writing current density may involve multiple origins such as the thermal-induced anisotropic expansion or the resistive switching^14–17^.

**Fig. 1:**
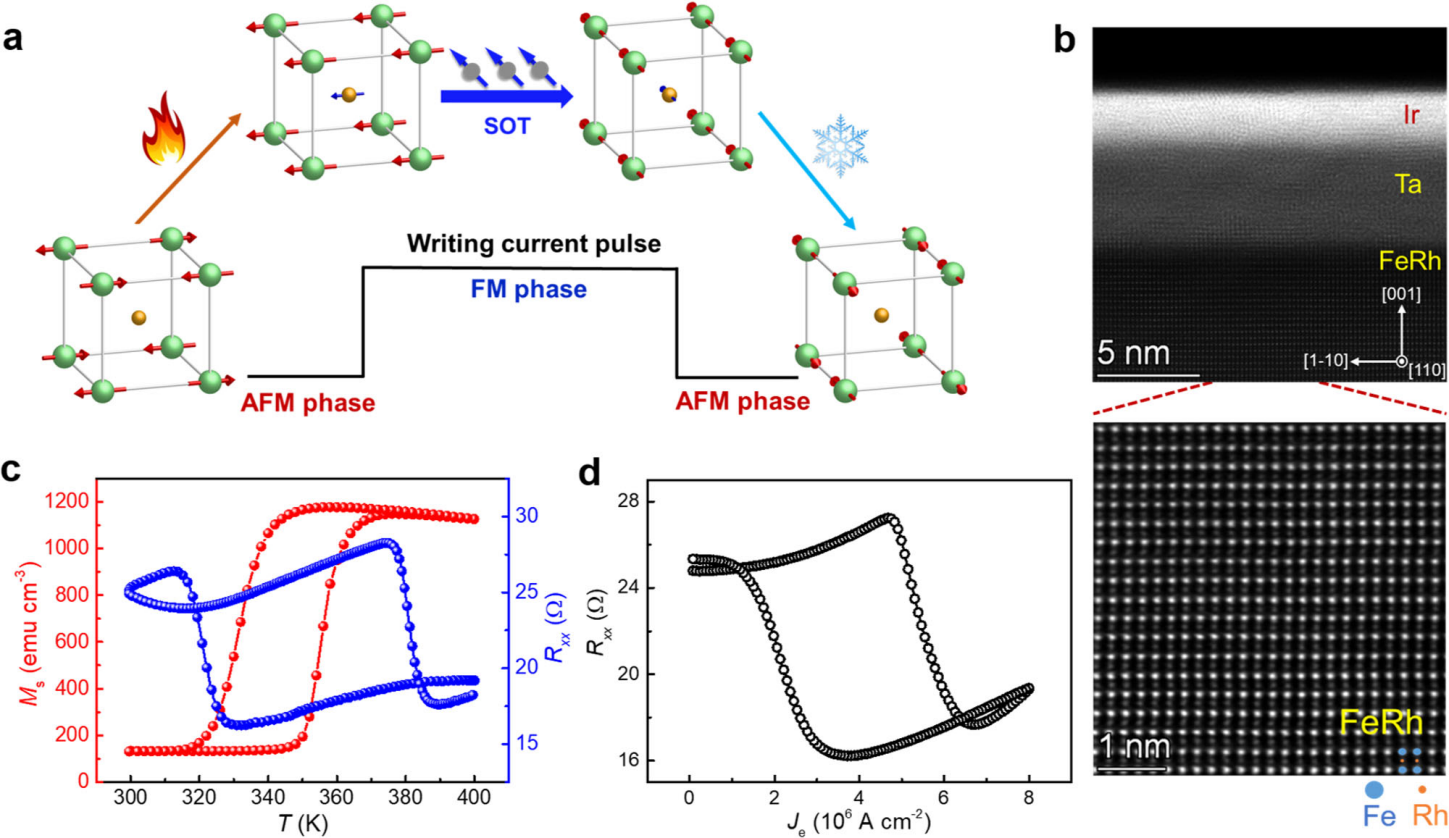
Schematic of the magnetic phase transition mediated Néel vector switching. **a** Schematic of the magnetic phase transition mediated Néel vector switching. Once a writing current pulse is applied, the Joule heating effect would induce the AFM-FM phase transition, and thus the spin-orbit torque (SOT) could efficiently switch the magnetic vector of the FM phase to the 90^o^ direction; when the writing pulse is off, another FM–AFM phase transition during the cooling process would print the 90^o^ switching from the FM phase (magnetic vector) to the AFM phase (Néel vector). **b** Cross-sectional transmission electron microscopy (TEM) results of FeRh/Ta/Ir heterostructures, where the FeRh film is along the (001) direction. **c** Temperature-driven AFM–FM phase transition, which is reflected by the saturation magnetization *M*_s_ and the longitudinal resistance *R*_*xx*_. **d** Current-induced Joule heating effect-driven magnetic phase transition between AFM and FM.

FeRh is a room temperature AFM with the CsCl-type crystal structure, where the unique reversible AFM–FM phase transition between 300 and 400 K makes it to be a promising candidate for memory applications at room temperature^19,22–25^. Here, the magnetic phase transition of FeRh is employed to amplify the efficiency of current-driven Néel vector switching: by introducing an intermediate FM phase during the writing current pulse, by the AFM–FM phase transition originating from the Joule heating effect, and thus the current-induced spin-orbit torque (SOT) can efficiently switch the FM vector; finally, once the writing current pulse is off, during the cooling process, another FM–AFM phase transition will print the switching of the magnetic vector (FM phase) to the Néel vector (AFM phase) (Fig. 1a). The critical switching current density *J*_c_ for the Néel vector switching (AFM) of FeRh is reduced to 10^6^–10^7^ A cm^−^^2^ at room temperature, i.e., at the same order of the magnetic vector switching (FM). By changing the base temperature, we prove that the magnetic phase transition mediated Néel vector switching has a much higher energy efficiency than the pure AFM phase. Besides the electrical transport measurement, the Néel vector switching of FeRh is further verified by the X-ray magnetic linear dichroism (XMLD) measurement. With continually changing the component of the FM phase from 0% (pure AFM) to 100% (pure FM) in AFM-coupled sublattices for macro-spin simulations, the *J*_c_ for the Néel vector switching of AFM is found to be significantly reduced by introducing an FM component.

## Results

### Structural and magnetic properties

Figure 1b shows the high-resolution transmission electron microscope results of the material stack FeRh(20)/Ta(5)/Ir(2) (thickness in nanometers) and the scanning TEM (STEM) results of FeRh, which shows the epitaxy growth of the FeRh film on the MgO(001) substrate and the crystal orientation of FeRh(001)[110]. The (001) orientation of the FeRh film is also confirmed by the X-ray diffraction (Supplementary Note 1). Ta is the strong spin–orbit coupling layer to provide the SOT to FeRh^26,27^, and the sharp interface between Ta and FeRh supports high interfacial spin transparency. The zoom-in figure of the FeRh region shows the perfect bcc-type unit cell, where the 1:1 Fe and Rh elements are periodically distributed in the crystal lattice.

Figure 1c shows the temperature-driven AFM–FM phase transition of FeRh, as indicated by the 1 order of magnitude increase of the magnetization beyond 370 K (*T*_AFM–FM_). The above room-temperature *T*_AFM–FM_ of FeRh sheds light on the potential of the electrical driven AFM–FM phase transition by the Joule heating effect. Compared to the AFM phase, the FM phase has a much higher density of states near the Fermi surface^28^, resulting in a pronounced low resistance state for the FM phase, which also provides a simple electrical way to detect the AFM–FM phase transition in the device level. Figure 1d shows that the current-induced Joule heating effect can efficiently drive the magnetic phase transition between AFM (high resistance) and FM (low resistance), with the order of 10^6^ A cm^−2^ current density, based on the electrical transport measurement in a 20 μm × 20 μm device.

### Current-driven Néel vector switching

**Fig. 2:**
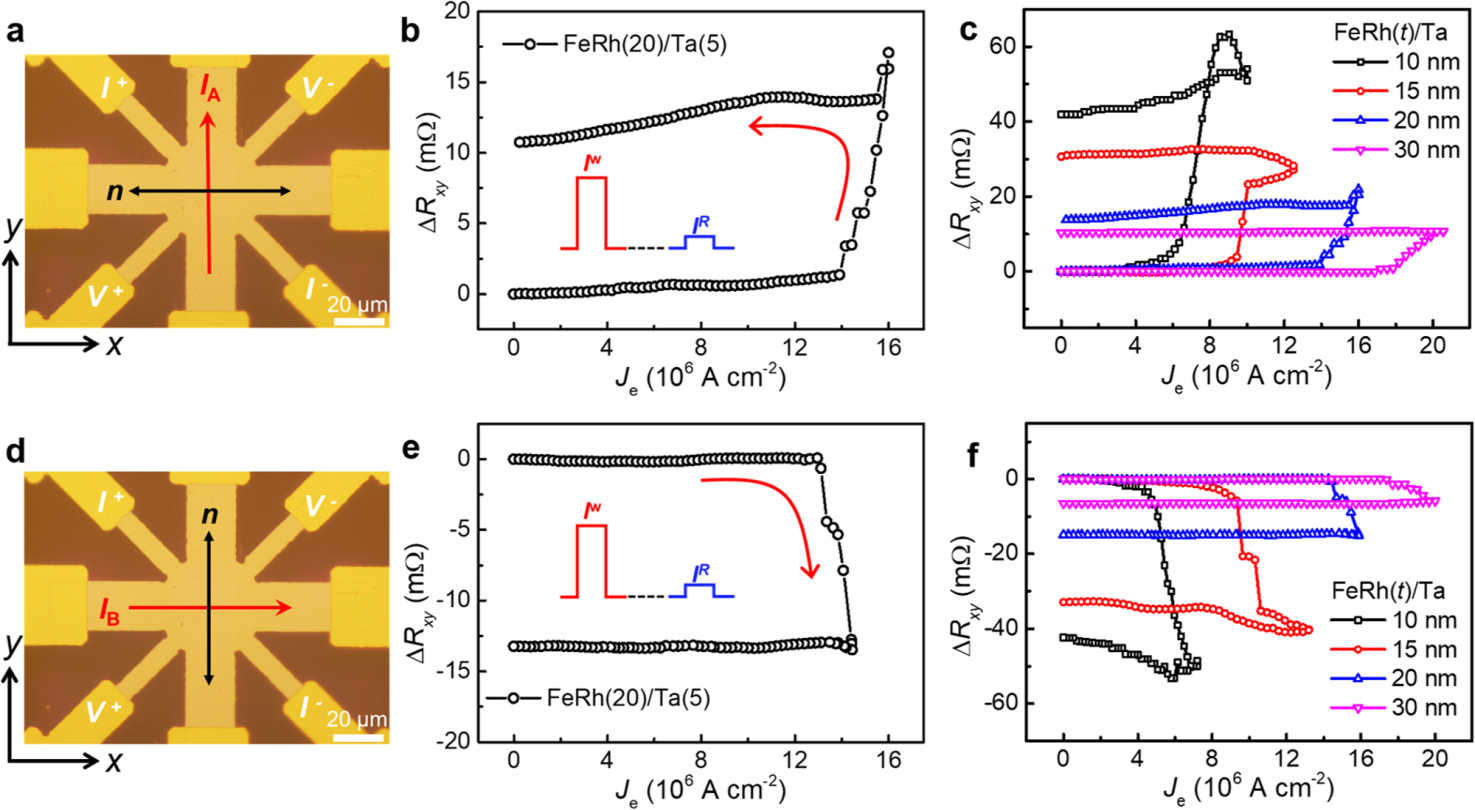
Current-driven Néel vector switching. **a**–**c**, **d**–**f** The current-driven Néel vector switching with the *y*-directional writing current *I*_A_ and the *x*-directional writing current *I*_B_, respectively, which switch the Néel vector to the *x*-axis and the *y* axis accordingly. **b**, **e** The planar Hall resistance Δ*R*_*xy*_ as a function of the writing current density *J*_e_, by the 2-pulses measurement, i.e., a writing current pulse *I*^w^ (*y*-direction) is applied to switch the Néel vector firstly, followed by a reading current pulse *I*^R^ in the −45^o^ channel to read the Néel vector by the planar Hall resistance (+45^o^ channel) at 1-s later. **c**, **f** The Δ*R*_*xy*_-*J*_e_ curves with the thickness of FeRh from 10 to 30 nm.

The 8-terminal device is employed to investigate the magnetic phase transition mediated Néel vector switching by the planar Hall measurement^29–32^, as shown in Fig. 2a. A 1-ms writing current pulse *I*_A_ is applied along the *y*-direction firstly, to switch the Néel vector ***n*** from the *y*-axis to the *x*-axis, which is detected by the orthogonal 1-ms reading current (−45^o^) by the planar Hall voltage (+45^o^) at 1-s later, i.e., 2-pulses measurement. The temporal *R*_*xx*_ measurement under the excitation of the 1-ms writing current pulse shows that the whole process of the Joule heating and cooling induced AFM–FM and FM–AFM phase transitions are within 1.2 ms (Supplementary Note 7), therefore, for the 1-s delay time here, the reading current detects the final stable AFM state. The time-resolved magneto-optic Kerr effect measurements have demonstrated a 100-ps speed of the AFM–FM transition and a 500-ps speed of the FM–AFM transition in FeRh^33–35^, inspiring the potential of the sub-ns speed of FeRh devices by ultrafast writing in the future. Figure 2b shows the Δ*R*_*xy*_-*J*_e_ curve of 2-pulses measurement for the FeRh(20)/Ta(5) structure, and the planar Hall resistance Δ*R*_*xy*_ only switches beyond the critical current density *J*_c_ of 1.4 × 10^7^ A cm^−2^ (i.e., threshold behavior, 6.0 × 10^6^ A cm^−2^ in the Ta layer) which is much larger than the current density *J*_p_ (6.8 × 10^6^ A cm^−2^) to induce the AFM-FM phase transition, indicating that the current-driven Néel vector switching here is indeed mediated by the magnetic phase transition. With further reducing the writing current density to zero, the non-volatile switching of Δ*R*_*xy*_ maintains, indicating the non-volatile Néel vector switching and excluding the high writing current density induced thermal effect. The current-driven Néel vector switching is robust for the FeRh thickness *t* from 10 to 30 nm, where the *J*_c_ gradually decreases with reducing *t*, down to 7.8 × 10^6^ A cm^−2^ (3.2 × 10^6^ A cm^−2^ in the Ta layer), as shown in Fig. 2c, indicating the interfacial (FeRh/Ta interface) spin current injection induced SOT. The step-like switching of Δ*R*_*xy*_ rather than the gradual change of Δ*R*_*xy*_ is also a signature of the Néel vector switching^16^. The current-induced Néel vector switching is further verified by the XMLD measurement (Supplementary Note 8 and Note 9).

Afterward, the writing current channel is changed from the *y*-direction to the *x*-direction (Fig. 2d), i.e., *I*_B_, and thus the Néel vector *n* of FeRh has switched back from the *x*-axis to the *y*-axis accordingly, where the reading configuration is the same, resulting in the planar Hall resistance Δ*R*_*xy*_ drop in Fig. 2e, which in contrast to the rising Δ*R*_*xy*_ (Néel vector switches from *y* to *x*) in Fig. 2b. The *J*_c_ for Néel vector switching from *y* to *x* and from *x* to *y* are symmetric, for all the samples with different FeRh thicknesses, as shown in Fig. 2f, c.

### Temperature dependence

**Fig. 3:**
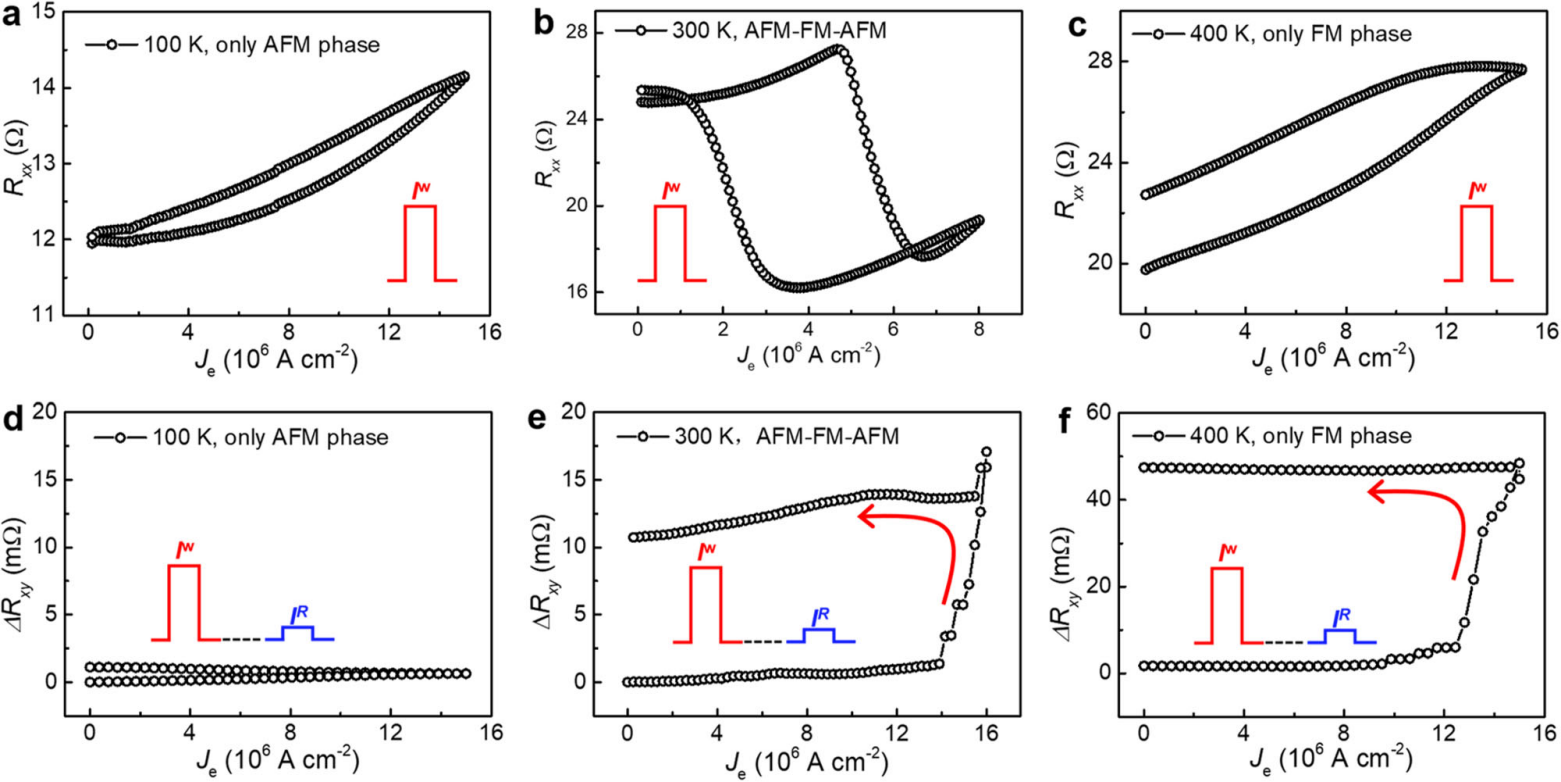
Temperature dependence of Néel vector switching. **a**–**c** Longitudinal resistance *R*_*xx*_ as a function of the writing current *J*_e_ (single pulse measurement) at a different base temperature of 100, 300, and 400 K, respectively; indicating that the only AFM phase at 100 K (**a**), AFM–FM–AFM phase transition at 300 K (**b**), and the only FM phase at 400 K (**c**), within the writing current density below 15 × 10^6^ A cm^−2^. **d**–**f** Current-driven Néel vector switching (2-pulses measurement) at 100 K (only AFM phase), 300 K (AFM–FM–AFM phase), and 400 K (only FM phase), respectively.

The magnetic phase transition of FeRh strongly depends on the temperature, therefore, the Néel vector switching for FeRh(20)/Ta(5) is performed with a different base temperature of 100, 300, and 400 K, respectively. For the 100 K base temperature, the Joule heating effect induced temperature increase for the current density we used is not enough to reach the *T*_AFM–FM_ (370 K), as indicated by the absence of the AFM–FM phase transition induced sharp *R*_*xx*_ change in Fig. 3a (single pulse measurement). For the pure AFM phase in this case, as shown in Fig. 3d, the writing current density *J*_e_ only induces some gradually increasing of Δ*R*_*xy*_ (2-pulses measurement) by thermal effect, where no step-like Néel vector switching happens due to the insensitivity to the SOT. At 300 K, the existence of the Joule heating induced AFM–FM phase transition (Fig. 3b) can efficiently mediate the Néel vector switching by the transient FM phase (Fig. 3e). For the 400 K beyond the AFM–FM phase transition temperature, the magnetic vector of the pure FM phase can also be switched by the writing current density *J*_e_ with a step-like behavior, with a much larger Δ*R*_*xy*_ compared to the AFM phase, as shown in Fig. 3c, f.

### Magnetic bias field dependence

**Fig. 4:**
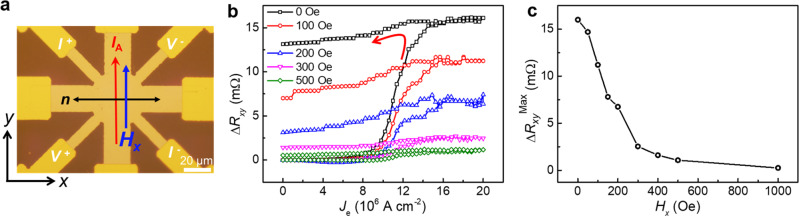
Magnetic bias field dependence of Néel vector switching. **a** Schematic of the magnetic bias field dependence measurement. An external magnetic bias field *H*_*x*_ along the writing current direction is applied during the current-driven Néel vector switching. **b** Current-driven Néel vector switching at different bias field *H*_*x*_. **c** Current-driven switching of the planar Hall resistance Δ*R*_*xy*_^Max^ as a function of *H*_*x*_.

Next, a series of external magnetic bias field *H*_*x*_ from 0 to 500 Oe is applied along the writing current direction during the Néel vector switching measurement, as shown in Fig. 4a and b. With increasing the magnitude of *H*_*x*_, the switching ratio of the Néel vector gradually reduces, where the *J*_c_ slightly increases at the same time. For FeRh(20)/Ta(5), during the writing current pulse, *H*_*x*_ would pin some magnetic domains of the transient FM phase after the Joule heating induced AFM–FM phase transition, by the way of additional Zeeman energy. As a result, once the writing pulse is off, the pinning of the magnetic vector of the transient FM state would be printed to the Néel vector of the final AFM phase, after the FM–AFM phase transition in the cooling process. Figure 4c shows the Δ*R*_*xy*_^Max^–*H*_*x*_ curve, and the Néel vector is totally pinned above 500 Oe, which is consistent with the saturation field of the transient FM phase (Supplementary Note 3).

### Macro-spin simulations

**Fig. 5:**
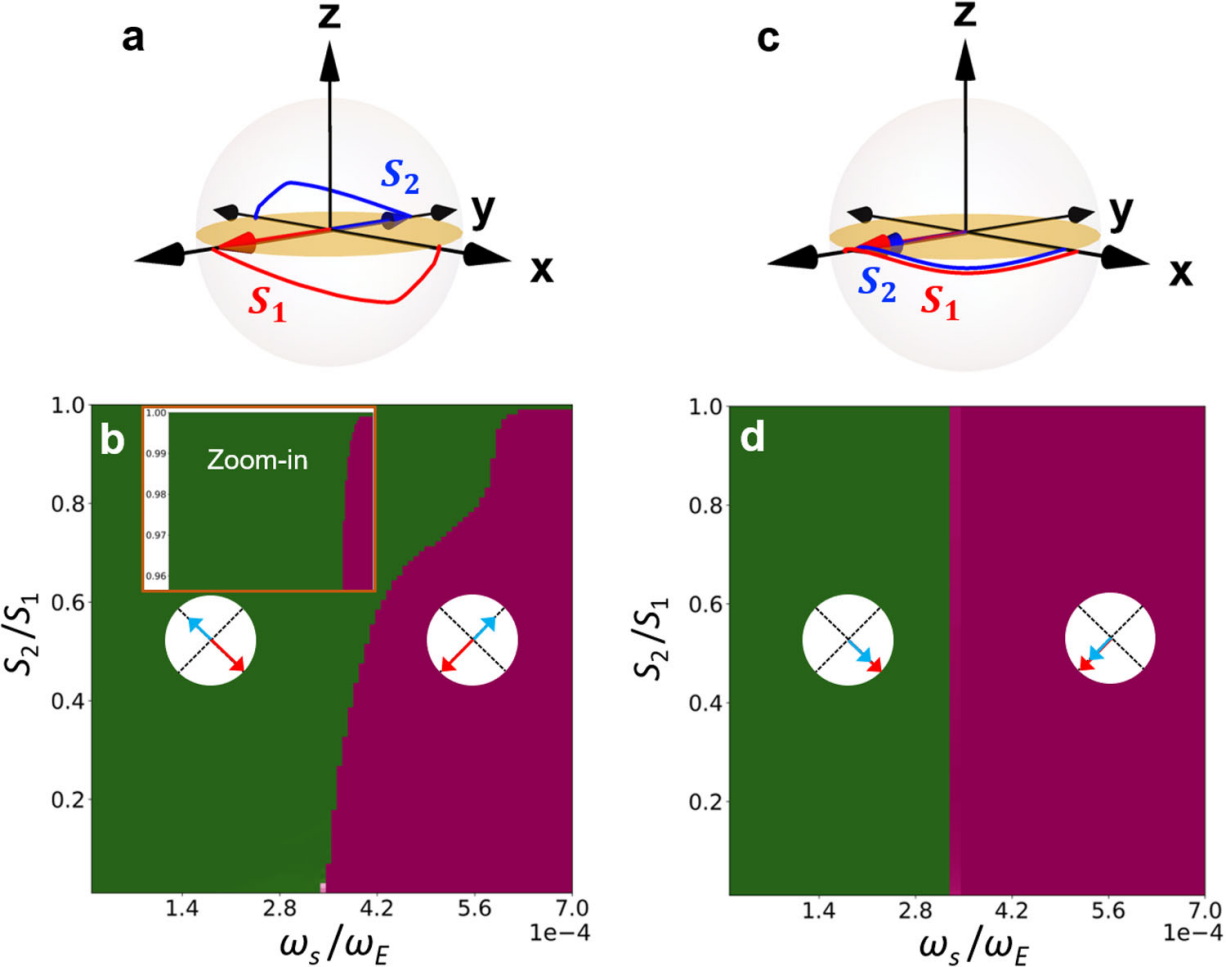
Trajectory and dynamical phase diagrams for SOT-induced switching. **a**, **c** Trajectories of antiparallel and parallel spins with ${S}_{2}/{S}_{1}=0.9$ and other parameters explained in “Methods”. **b**, **d** Dynamical phase diagrams in terms of the terminal state of the order parameter [Néel vector ${{{\boldsymbol{n}}}}$ in (**b**) and magnetic vector ${{{\boldsymbol{m}}}}$ in (**d**)] plotted for ${S}_{2}/{S}_{1}\in [{{\mathrm{0,1}}}]$ with ${S}_{1}+{S}_{2}=2$. The unswitched and switched regions are marked by different colors and illustrated by the insets.

Using macro-spin simulations^36^, we can uncover the crucial role of the transient FM state in facilitating the Néel vector switching by comparing the switching thresholds for the AFM and FM phases under the same materials parameters. Even though a macro-spin study cannot reproduce the actual threshold in a multi-domain process, it suffices to reveal the essential difference of the SOT-induced switching between the AFM and FM phases (see “Methods” for detailed algorithms). As shown in Fig. 5, we continuously vary the ratio of the two sublattice spins $\frac{{S}_{2}}{{S}_{1}}$ from $0$ to $1$ while keeping the total spin ${S}_{1}+{S}_{2}$ constant. As shown in Fig. 5a, a representative switching trajectory in the AFM phase (antiparallel coupling) involves significant out-of-plane motion, while in the FM phase (parallel coupling) the switching process is essentially an in-plane rotation as shown in Fig. 5c. In the AFM phase, as shown in Fig. 5b, the switching threshold increases monotonically with an increasing $\frac{{S}_{2}}{{S}_{1}}$ and eventually diverges in the compensation limit $\frac{{S}_{2}}{{S}_{1}}\to 1$. By contrast, the switching threshold in the FM phase remains a constant independent of $\frac{{S}_{2}}{{S}_{1}}$, as shown in Fig. 5d. The switching thresholds of the two phases approach the same in the limit $\frac{{S}_{2}}{{S}_{1}}\to 0$ (*i.e*., one sublattice spin vanishes).

To achieve an intuitive understanding of the distinct behavior shown above, we now derive effective models of the order parameter dynamics for the two phases. Using the unit vectors ${\hat{{{{\boldsymbol{S}}}}}}_{{{{\boldsymbol{1}}}}}={{{{\boldsymbol{S}}}}}_{{{{\boldsymbol{1}}}}}{{{\boldsymbol{/}}}}{S}_{1}$ and ${\hat{{{{\boldsymbol{S}}}}}}_{{{{\boldsymbol{2}}}}}\,{{{\boldsymbol{=}}}}\,{{{{\boldsymbol{S}}}}}_{{{{\boldsymbol{2}}}}}{{{\boldsymbol{/}}}}{S}_{2}$, we define the Néel vector ${{{\boldsymbol{n}}}}\,{{{\boldsymbol{=}}}}\,\left({\hat{{{{\boldsymbol{S}}}}}}_{{{{\boldsymbol{1}}}}}-{\hat{{{{\boldsymbol{S}}}}}}_{{{{\boldsymbol{2}}}}}\right)/2$ and the magnetic vector ${{{\boldsymbol{m}}}}\,{{{\boldsymbol{=}}}}\,\left({\hat{{{{\boldsymbol{S}}}}}}_{{{{\boldsymbol{1}}}}}+{\hat{{{{\boldsymbol{S}}}}}}_{{{{\boldsymbol{2}}}}}\right)/2$, which satisfies ${{{\boldsymbol{n}}}}{{{\boldsymbol{\cdot }}}}{{{\boldsymbol{m}}}}\,{{{\boldsymbol{=}}}}\,0$ and ${\left|{{{\boldsymbol{n}}}}\right|}^{{{{\boldsymbol{2}}}}}+{\left|{{{\boldsymbol{m}}}}\right|}^{{{{\boldsymbol{2}}}}}{{{\boldsymbol{=}}}}1$. In the exchange limit, ${\hat{{{{\boldsymbol{S}}}}}}_{{{{\boldsymbol{1}}}}}$ and ${\hat{{{{\boldsymbol{S}}}}}}_{{{{\boldsymbol{2}}}}}$ are almost antiparallel (parallel) during the switching process such that the AFM (FM) phase is characterized by ${{{\boldsymbol{n}}}}$ (${{{\boldsymbol{m}}}}$) since $\left|{{{\boldsymbol{n}}}}\right|\approx 1$ and $\left|{{{\boldsymbol{m}}}}\right|\; < < \;{{{\boldsymbol{\ll }}}}1$ ($\left|{{{\boldsymbol{m}}}}\right|\approx 1$ and $\left|{{{\boldsymbol{n}}}}\right| < < {{{\boldsymbol{\ll }}}}1$). In the AFM phase, we can eliminate ${{{\boldsymbol{m}}}}$ from the coupled Landau–Lifshitz–Gilbert (LLG) equations for ${{{{\boldsymbol{S}}}}}_{{{{\boldsymbol{1}}}}}$ and ${{{{\boldsymbol{S}}}}}_{{{{\boldsymbol{2}}}}}$ and obtain an effective equation for ${{{\boldsymbol{n}}}}$ alone

1 $$	\ddot{{{{\boldsymbol{n}}}}}+{\omega }_{E}\left({S}_{1}-{S}_{2}\right){{{\boldsymbol{n}}}}\times \dot{{{{\boldsymbol{n}}}}}+\alpha S{\omega }_{E}\dot{{{{\boldsymbol{n}}}}}\\ 	=\frac{{\omega }_{E}{S}^{2}}{2{S}_{1}{S}_{2}}\left[{\omega }_{\parallel }\left({n}_{x}^{2}-{n}_{y}^{2}\right)\left({n}_{x}\hat{{{{\boldsymbol{x}}}}}-{n}_{y}\hat{{{{\boldsymbol{y}}}}}\right)-{\omega }_{\perp }{n}_{z}\hat{{{{\boldsymbol{z}}}}}-{\omega }_{s}{{{\boldsymbol{p}}}}\times {{{\boldsymbol{n}}}}\right]$$

where ${\omega }_{E}$ is the exchange interaction, ${\omega }_{\perp }$ is the hard axis anisotropy along $z$-axis, and ${\omega }_{\parallel }$ is the fourfold easy anisotropy in the ${xy}$ plane, all scaled into angular frequencies. Here, $\alpha$ is the Gilbert damping, $S={S}_{1}+{S}_{2}={{{\rm{const}}}}.$, $\hat{{{{\boldsymbol{x}}}}}$, $\hat{{{{\boldsymbol{y}}}}}$, and $\hat{{{{\boldsymbol{z}}}}}$ are the unit vectors of the Cartesian coordinates, ${\omega }_{s}$ and ${{{\boldsymbol{p}}}}$ are the strength and the polarization direction of the SOT, respectively. Equation (1) can explain the change of switching threshold with a varying $\frac{{S}_{2}}{{S}_{1}}$ and the out-of-plane motion of ${{{\boldsymbol{n}}}}$ shown in Fig. 5a and b. Suppose that initially ${{{\boldsymbol{n}}}}$ is along $\hat{{{{\boldsymbol{x}}}}}$; ${{{\boldsymbol{p}}}}$ is along $-\hat{{{{\boldsymbol{y}}}}}$. The SOT term ${\omega }_{s}{{{\boldsymbol{p}}}}\times {{{\boldsymbol{n}}}}$ perpetuals ${{{\boldsymbol{n}}}}$ towards the $-z$ direction, so $\dot{{{{\boldsymbol{n}}}}}$ acquires a $z$ component. In the compensation limit ${S}_{1}={S}_{2}$, the second term ${\omega }_{E}\left({S}_{1}-{S}_{2}\right){{{\boldsymbol{n}}}}\times \dot{{{{\boldsymbol{n}}}}}$ vanishes identically, so the motion of ${{{\boldsymbol{n}}}}$ is restricted to the ${xz}$ plane because no other term in Eq. (1) has $y$ component. That is to say, an SOT with ${{{\boldsymbol{p}}}}$ along $\pm\! y$ can only tilt the order parameter towards $\pm\! z$ but cannot push it away from the ${xz}$ plane. Consequently, an in-plane switching process cannot be initiated, explaining the diverging threshold. As we move away from the compensation limit, however, the term ${\omega }_{E}\left({S}_{1}-{S}_{2}\right){{{\boldsymbol{n}}}}\times \dot{{{{\boldsymbol{n}}}}}$ emerges rapidly since ${\omega }_{E}$ is extremely large. It is this term that generates a $y$ component in Eq. (1), triggering a jump of ${{{\boldsymbol{n}}}}$ out of the ${xz}$ plane, which subsequently induces a switching process. This well explains the switching trajectory shown in Fig. 5a, where an initial motion along $z$ suddenly becomes a rotation in the ${xy}$ plane. Since this crucial term is proportional to the spin imbalance $\left({S}_{1}-{S}_{2}\right)$, it also explains why the switching threshold decreases as we move towards smaller ${S}_{2}/{S}_{1}$, as shown in Fig. 5b. The zoom-in figure shows the discontinuous transition from no switching to a finite switching with a threshold current, which comes from the order parameter (${{{\boldsymbol{m}}}}$) changing from zero to a finite value. The exact AFM system has a different symmetry compared to the proposed Néel vector+magnetic vector (i.e., ***n*** + ***m***) system, even though the ***m*** component is infinitely small. With a very tiny effective magnetic field (such as from current-induced SOT) to break the (inversion) symmetry of the Néel vector, the discontinuous behavior is removed, which is the real case of this work.

Following a similar procedure, we can derive the effective dynamics ${{{\boldsymbol{m}}}}$ for the FM phase by eliminating ${{{\boldsymbol{n}}}}$

2 $$\dot{{{{\boldsymbol{m}}}}}=\frac{S}{2{S}_{1}{S}_{2}}{{{\boldsymbol{m}}}}\times \left[{\omega }_{\perp }{m}_{z}\hat{{{{\boldsymbol{z}}}}}-{\omega }_{\parallel }\left({m}_{x}^{2}-{m}_{y}^{2}\right)\left({m}_{x}\hat{{{{\boldsymbol{x}}}}}-{m}_{y}\hat{{{{\boldsymbol{y}}}}}\right)+{\omega }_{s}\left({{{\boldsymbol{p}}}}\times {{{\boldsymbol{m}}}}\right)\right]+\alpha {{{\boldsymbol{m}}}}\times \dot{{{{\boldsymbol{m}}}}}$$

where all parameters are defined in the same way as the AFM case. Equation (2) differs from Eq. (1) since the second-order time derivative disappears. The order parameter ${{{\boldsymbol{m}}}}$ simply follows the dynamics of an FM as if there is no sublattice. In Eq. (2), both the anisotropy torques and the SOT scale with a common factor $\frac{S}{2{S}_{1}{S}_{2}}$. Therefore, their competition is independent of $\frac{{S}_{2}}{{S}_{1}}$, so the switching threshold is constant with changing $\frac{{S}_{2}}{{S}_{1}}$, as shown in Fig. 5d. Also distinct from the AFM phase, the SOT term ${\omega }_{s}{{{\boldsymbol{m}}}}\times \left({{{\boldsymbol{p}}}}\times {{{\boldsymbol{m}}}}\right)$ now only generates an in-plane torque acting on ${{{\boldsymbol{m}}}}$, which does not lead to an out-of-plane motion, consistent with the trajectory in Fig. 5c.

## Discussion

In conclusion, the current-driven Néel vector switching is realized in FeRh/Ta, by the Joule heating-induced magnetic phase transition (between AFM and FM) and the SOT simultaneously. The participation of the transient FM phase during the writing current significantly reduces the switching current density *J*_c_ of the Néel vector, as indicated by both the experiment and simulation results. The Néel vector switching is further verified by the temperature and magnetic bias field dependences, and the XMLD results. Compared to the previous work of the FeRh memory based on the conventional field-cooling process^22^, the all-electrical operation in our work makes a crucial and practical step for the high-density memory applications. This work provides a heat-assisted approach for the efficient electrical manipulation of the antiferromagnetic order, by the Joule heating induced magnetic phase transition, which helps to reduce the writing current density for future AFM devices.

## Methods

### Sample growth and device fabrication

Epitaxial equiatomic FeRh thin films were deposited on commercial (100)-oriented MgO substrates in a magnetron sputtering system with a base pressure of below 1.0 × 10^−8^ Torr. Prior to deposition, the substrates were annealed at 530 °C for 1 h in a vacuum chamber. The film was grown at a temperature of 530 °C and then annealed at 670 °C for 1 h. The film thicknesses were controlled based on the deposition time, which was calibrated through the X-ray reflectivity. The Ta(5)/Ir(2) layers were grown on top of the FeRh by the magnetron sputtering system, and a 120 s pre-etching was performed to clean the surface of FeRh (etch about 1–2 nm). The FeRh/Ta/Ir heterostructures were patterned into the 8-terminal and Hall-bar devices by the standard photolithography combined with the dry etching method.

### Magnetic and spin transport measurements

The spin-transport properties were measured by the probe station system with an electromagnet and the physical property measurement system, where the pulse current was applied by the Keithley 2612 current source, and the voltage signals were measured by the Keithley 2182A nano voltmeter. The magnetic properties of FeRh were measured by the superconducting quantum interference device (SQUID) system.

### Macro-spin simulations

Since all spins originate from the Fe^2+^ ions, we use the same gyromagnetic ratio $\gamma$ for both sublattices. Scaling all interactions into angular frequencies that absorbe the $\gamma$ factor, we can express the free energy as

3 $$F/{{\hslash }}={\omega }_{E}{{{{\boldsymbol{S}}}}}_{1}\cdot {{{{\boldsymbol{S}}}}}_{2}+\mathop{\sum }\limits_{i=1}^{2}\left\{\frac{{\omega }_{\perp }}{2{S}_{i}^{2}}{S}_{{iz}}^{2}-\frac{{\omega }_{\parallel }}{4{S}_{i}^{4}}{\left({S}_{{ix}}^{2}-{S}_{{iy}}^{2}\right)}^{2}\right\}$$

where ${\omega }_{E}$ is positive (negative) for antiparallel (parallel) spin configurations, ${\omega }_{\perp }$ and ${\omega }_{\parallel }$ are defined in the main text, ${S}_{{ix}}$, ${S}_{{iy}}$, and ${S}_{{iz}}$ are the projections of ${{{{\boldsymbol{S}}}}}_{i}$ on the Cartesian axes. In both the AFM and the FM phases, the spin dynamics is simulated by solving the coupled LLG equations for each sublattice spin

4 $${\dot{{{{\boldsymbol{S}}}}}}_{i}={{{{\boldsymbol{h}}}}}_{i}\times {{{{\boldsymbol{S}}}}}_{i}+\frac{\alpha }{{S}_{i}}{{{{\boldsymbol{S}}}}}_{i}\times {\dot{{{{\boldsymbol{S}}}}}}_{i}+\frac{{\omega }_{s}}{{S}_{i}^{2}}{{{{\boldsymbol{S}}}}}_{i}\times \left({{{\boldsymbol{p}}}}\times {{{{\boldsymbol{S}}}}}_{i}\right),\,i=1,2$$

where ${{{{\boldsymbol{h}}}}}_{i}=-\delta F/\hslash \delta {{{{\boldsymbol{S}}}}}_{i}$ is the effective field acting on ${{{{\boldsymbol{S}}}}}_{i}$, $\alpha$ is the Gilbert damping, ${\omega }_{s}$ and ${{{\boldsymbol{p}}}}$ are the strength and spin polarization direction of the SOT, respectively. In performing the simulation, we fix the total spin ${S}_{1}+{S}_{2}=2$ while varying the ratio $\frac{{S}_{2}}{{S}_{1}}$ from $0$ to $1$. Basing on typical materials parameters, we set $\frac{\left|{\omega }_{E}\right|}{2\pi }=10$ THz, $\frac{{\omega }_{\perp }}{2\pi }=20$ GHz, $\frac{{\omega }_{\parallel }}{2\pi }=1$ GHz, and $\alpha =0.05$. We have chosen ${{{\boldsymbol{p}}}}\,{{{\boldsymbol{=}}}}\;{{{\boldsymbol{-}}}}\hat{{{{\boldsymbol{y}}}}}$ so that the initial condition is ${{{{\boldsymbol{S}}}}}_{i}\left(0\right)={\left(-1\right)}^{i-1}{S}_{i}\hat{{{{\boldsymbol{x}}}}}$. The dynamical phase diagrams are plotted for the terminal state, i.e., when both spin sublattices have fully relaxed to equilibrium after the application of the SOT.

## Supplementary information

Supplementary Information

## Data Availability

The authors declare that the main data supporting the findings of this study are available within the article and its Supplementary Information files. Extra data are available from the corresponding author upon reasonable request.
