# Supplementary material for: Current-induced Néel order switching facilitated by magnetic phase transition
Source: Nat Commun. 2022 Mar 28;13:1629. doi: 10.1038/s41467-022-29170-2 (PMC8960908; doi:10.1038/s41467-022-29170-2)
Supplement: Supplementary file 1 — Supplementary Information [file 41467_2022_29170_MOESM1_ESM.pdf]

Supplementary Information

Current-induced Néel order switching facilitated by magnetic phase transition

Hao Wu[1,2#*], Hantao Zhang[3#], Baomin Wang[4,5*], Felix Groß[6], Chao-Yao Yang[7], Gengfei Li[5], Chenyang Guo[8], Haoran He[1], Kin Wong[1], Di Wu[1], Xiufeng Han[2,8], Chih-Huang Lai[7], Joachim Gräfe[6], Ran Cheng[3*], and Kang L. Wang[1*]

[1]*Department of Electrical and Computer Engineering, University of California, Los Angeles, California 90095, USA*

[2]*Songshan Lake Materials Laboratory, Dongguan, Guangdong 523808, China*

[3]*Department of Electrical and Computer Engineering, University of California, Riverside, California 92521, USA*

[4]*School of Physical Science and Technology, Ningbo University, Ningbo 315211, China*

[5]*CAS Key Laboratory of Magnetic Materials and Devices, Ningbo Institute of Materials Technology and Engineering, Chinese Academy of Sciences, Ningbo 315201, China*

[6]*Max Planck Institute for Intelligent Systems, Heisenbergstraße 3, Stuttgart 70569, Germany*

[7]*Department of Materials Science and Engineering, National Tsing Hua University, Hsinchu 30013, Taiwan*

[8]*Beijing National Laboratory for Condensed Matter Physics, Institute of Physics,*

Chinese Academy of Sciences, Beijing 100190, China

[#]These authors contributed equally to this work.

*Corresponding author. E-mail: wuhaoiphy@gmail.com; wangbaomin@nimte.ac.cn;

rancheng@ucr.edu; wang@ee.ucla.edu

Supplementary Note 1: XRD of the FeRh film.

The X-ray diffraction (XRD) is employed to analysis the crystal structure of the FeRh film on the MgO(001) substrate, with the thickness varies from 5 nm to 30 nm. The θ–2θ patterns in Supplementary Fig. 1a show the (001) orientation of the FeRh film, and the Laue oscillations in Supplementary Fig. 1b indicate the high-quality epitaxy growth of FeRh on the MgO substrate.

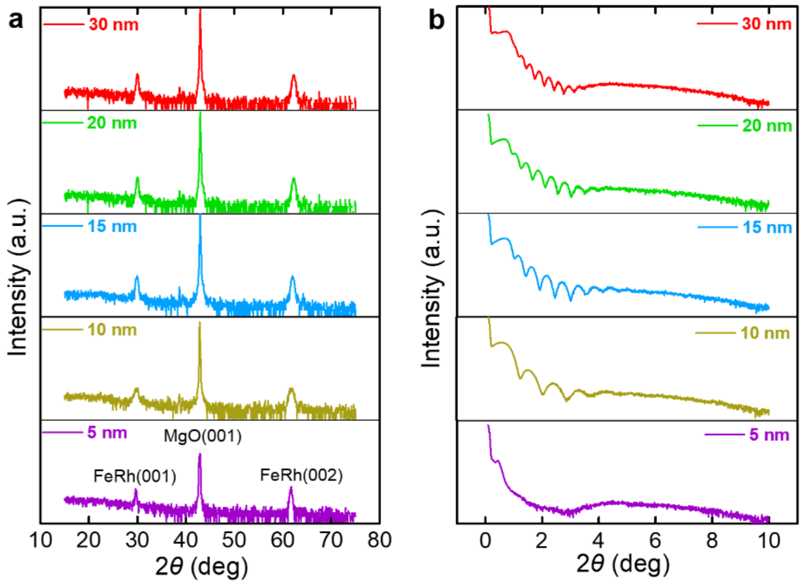

Supplementary Fig. 1: **XRD results. a**, XRD θ–2θ scan of MgO(001)/FeRh(t) heterostructures, with t from 5 nm to 30 nm. **b**, Laue oscillations indicate an epitaxy growth of the FeRh film on the MgO substrate.

Supplementary Note 2: HAADF and EDS mapping of the FeRh/Ta/Ir heterostructures.

Supplementary Fig. 2 a-e shows the high-angle annular dark field (HAADF) and the energy dispersive spectroscopy (EDS) mapping of the FeRh(20)/Ta(5)/Ir(2) (thickness

in nanometers) multilayer stack, where the sharp interface between Ta and FeRh supports a high interfacial spin transparency.

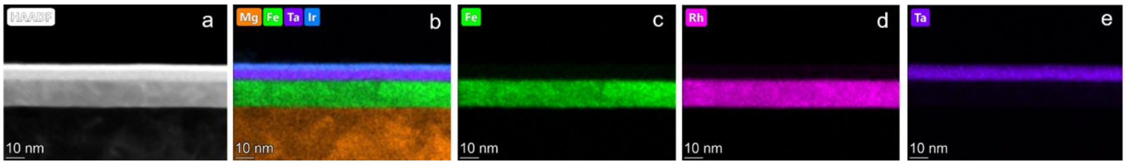

Supplementary Fig. 2: TEM results. a-e, HAADF and EDS mapping of the FeRh/Ta/Ir heterostructures.

Supplementary Note 3: *M-H* loops of the AFM phase and the FM phase.

For FeRh(t)/Ta heterostructures with t from 10 nm to 30 nm, the *M-H* loops of the AFM phase and the FM phase are measured at 300 K and 400 K, respectively, as shown in Supplementary Fig. 3a and 3b, where the saturation field H_s for AFM and FM are 2.0 kOe and 0.5 kOe, respectively.

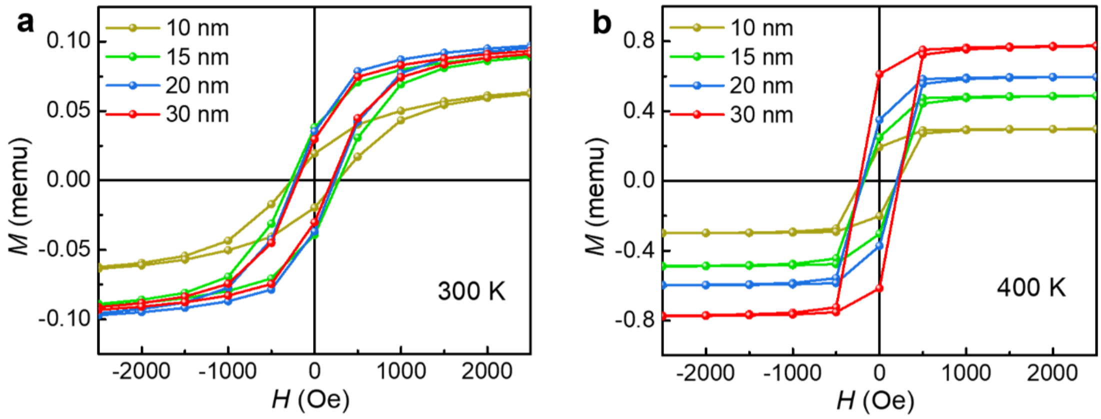

Supplementary Fig. 3: Magnetic loops. *M-H* loops measured at 300 K (AFM phase) (**a**) and 400 K (FM phase) (**b**), respectively.

Supplementary Note 4: Temperature and current driven magnetic phase transition.

The AFM-FM magnetic phase transition is measured by the longitudinal resistance R_{xx} change at the device level, for FeRh(t)/Ta heterostructures with t from 10 nm to 30 nm,

by the temperature and current-induced Joule heating, as shown in Supplementary Fig. 4a and 4b, respectively.

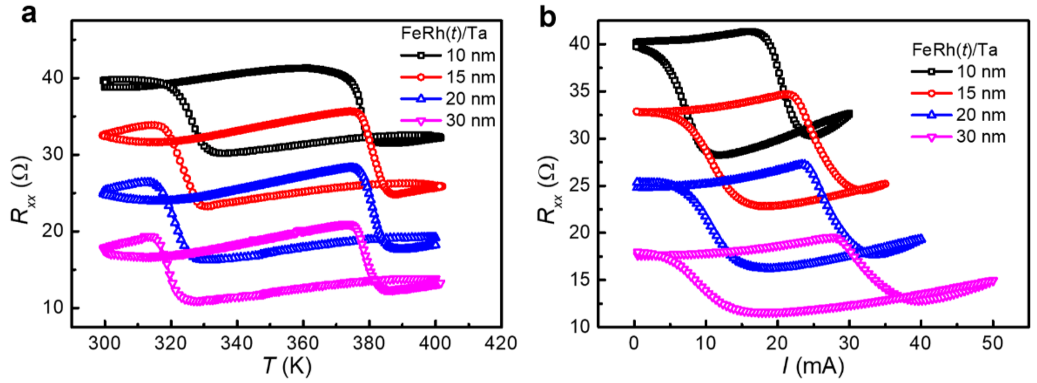

Supplementary Fig. 4: Transport measurement for AFM-FM transition. The temperature (**a**) and current (**b**) driven AFM-FM magnetic phase transition, measured by the change of the longitudinal resistance R_{xx}.

Supplementary Note 5: Angular dependence of the planar Hall resistance.

The angular dependence of the planar Hall resistance R_{xy}-θ in FeRh/Ta is measured at the base temperature of 300 K (AFM phase) and 400 K (FM phase), respectively, as shown in Supplementary Fig. 5a and 5b, with the sin2θ fitting, where a 5 kOe magnetic field is applied during the sample rotation. θ scans along the film plane, and 0° represents the current direction.

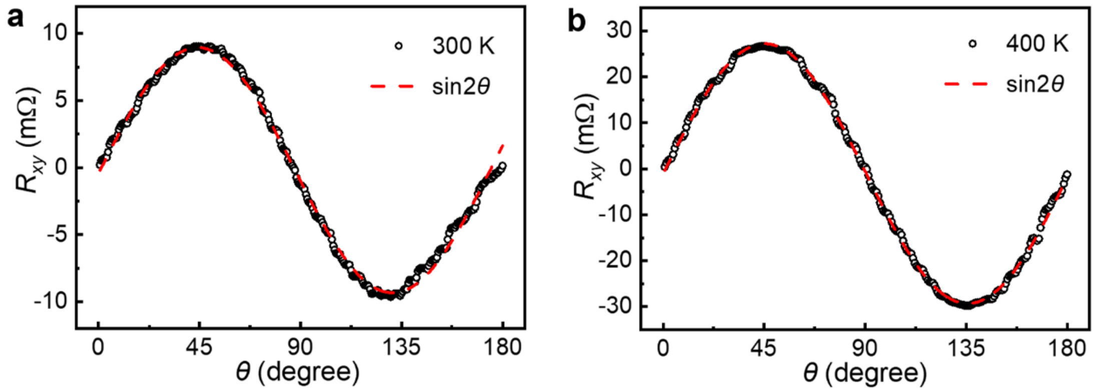

Supplementary Fig. 5: Angular dependence of planar Hall. a and **b** show the angular dependence of the planar Hall resistance in FeRh/Ta measured at 300 K (AFM phase) and 400 K (FM phase), respectively.

Supplementary Note 6: Current-driven Néel vector switching in FeRh/Pt.

Pt has the opposite sign of spin Hall angle with that in Ta[1], therefore, for comparison, we demonstrate the current-driven Néel vector switching in FeRh(15)/Pt(5) structures. For the current-driven 90° Néel vector switching, the different signs of spin Hall angle give rise to the same switching behavior, as shown in Supplementary Fig. 6, because the Néel vector is a uniaxial vector, therefore, θ° and (θ+180)° spin polarizations have no difference.

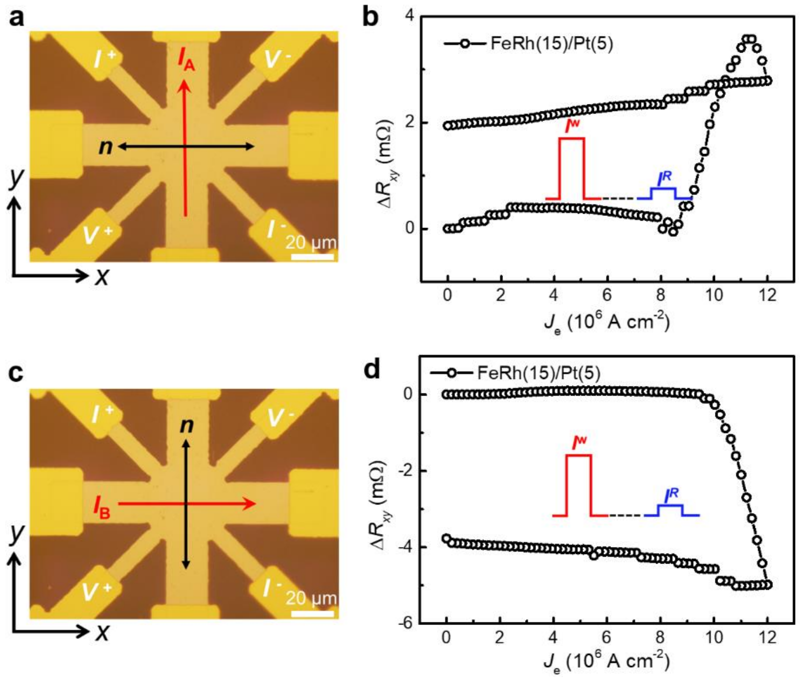

Supplementary Fig. 6: Current-driven Néel vector switching in FeRh/Pt. a and **b** show the current-driven 90° Néel vector switching with the y-directional writing current I_A. **c** and **d** show the results with the x-directional writing current I_B.

Supplementary Note 7: Temporal profile of the current-induced Joule heating and

cooling process.

Supplementary Fig. 7a and 7b show the temporal profile of the 1-ms writing current pulse and the longitudinal resistance R_{xx}, respectively, where a 2-mA DC bias current is applied to read the R_{xx} continuously during the measurement. The rising and falling time of the writing current pulse is 30 μs and 200 μs, respectively. From the high-low resistance state transition, we can get the 500 μs time scale of the Joule heating induced AFM-FM phase transition, which is mainly from the thermal accumulation process by Joule heating. When the writing current pulse is off, the cooling process induced FM-AFM phase transition (low-high resistance state transition) takes about 100 μs, which is mainly governed by the long falling time (200 μs) of the writing current pulse. These results indicate that the 1-s delay time between reading and writing in our manuscript is long enough for the Joule heating and the cooling induced AFM-FM-AFM phase transition, therefore, in the 2-pulses measurement, we measure the final stable AFM state, rather than the transient FM state.

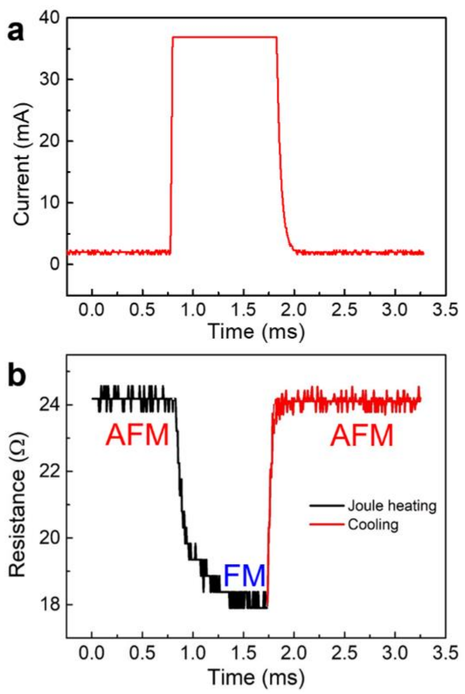

Supplementary Fig. 7: Temporal measurement. The temporal profile of the 1-ms writing current pulse (**a**) and the longitudinal resistance R_{xx} (**b**).

Supplementary Note 8: XMLD spectrum for the Néel vector switching.

The X-ray magnetic linear dichroism (XMLD) measurement is performed in the FeRh(10)/Ta sample, where the differential absorption of horizontal and vertical linear polarized X-ray (LH and LV) is employed to detect the Néel vector direction. Supplementary Fig. 8a and 8b show the X-ray absorption spectroscopy (XAS) and XMLD spectrum at Fe L_3 and L_2 edges for the initial state and the final state after a 1-ms writing current pulse (8×10^6 A cm^{-2}), and clearly the Néel vector is switched to the vertical direction with the writing current, indicating the SOT-type Néel vector switching.

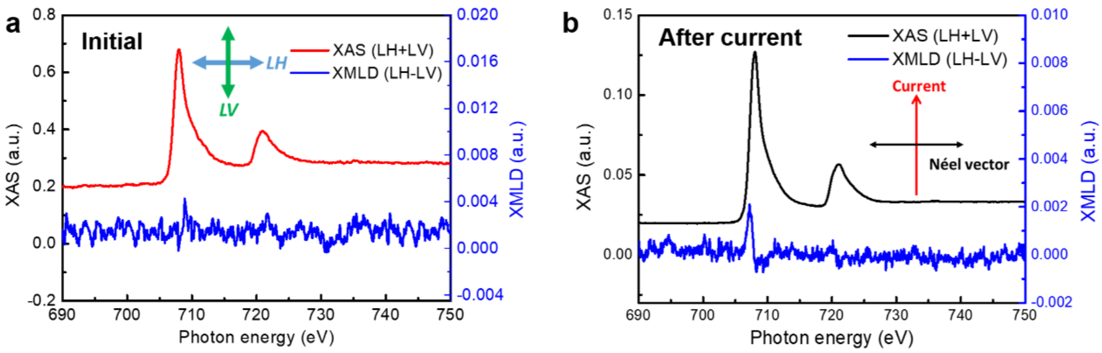

Supplementary Fig. 8: XMLD spectrum. a and **b** show the XAS and XMLD spectrums for the initial state and the final state after a 1-ms writing current pulse, respectively.

Supplementary Note 9: Antiferromagnetic domain switching.

The scanning transmission X-ray microscope (STXM) based on XMLD is employed to image the AFM domains and the current-driven AFM domains switching in FeRh(15)/Ta(5)[2-6]. Supplementary Fig. 9a and 9b show the initial AFM domains and the final AFM domains after a 1-s writing current pulse of 15 mA (7.5×10^6 A cm^{-2}, 10 μm width of the writing channel), where some parts of the AFM domains are switched by the writing current. The difference between the final (Supplementary Fig. 9b) and initial (Supplementary Fig. 9a) states is shown in Supplementary Fig. 9c, indicating the current-driven switching part of AFM domains. The limitation of the switching ratio of AFM domains here comes from the limited current density that can be applied in this fragile device which is thinning down below 100 nm for X-ray transmission.

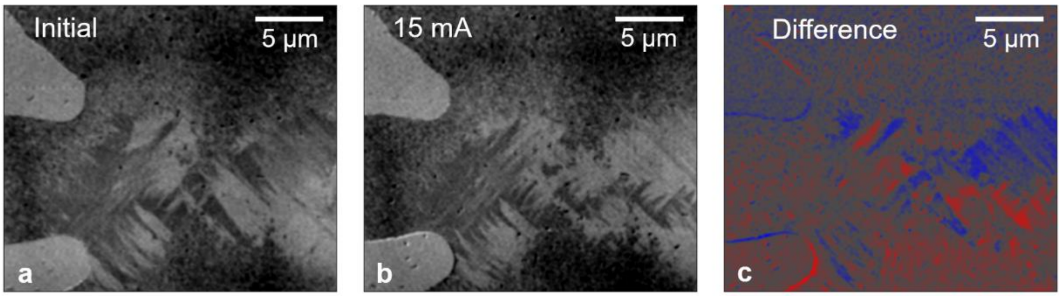

Supplementary Fig. 9: AFM domains measured by the scanning transmission X-ray microscope (STXM) based on XMLD. a and **b** show the AFM domains at the initial state and the final state after a 15-mA current pulse, respectively. **c**, The difference between the final and initial states shows the current-driven switching part of AFM domains.

Supplementary Note 10: Estimation of current distribution in FeRh/Ta/Ir.

Supplementary Table 1: Current distribution.

Layer	FeRh	Ta	Ir
Resistivity (μΩ cm)	125	292	49
Thickness (nm)	10	5	2
Current distribution	58%	12%	30%
Thickness (nm)	20	5	2
Current distribution	74%	8%	18%
Thickness (nm)	30	5	2
Current distribution	80%	6%	14%

Supplementary Note 11: Waiting time measurement.

After applying a 1-ms writing current pulse $J_e = 1.5 \times 10^7$ A cm^{-2}, we have read the ΔR_{xy} every 1 s from 1 s to 30 s by the 1-ms reading current pulse ($J_R = 1.9 \times 10^5$ A cm^{-2}), as shown in Supplementary Fig. 10, and the data show that the change of ΔR_{xy} is below 1% during 1 s to 30 s, indicating the heating artifacts are not prominent in our work.

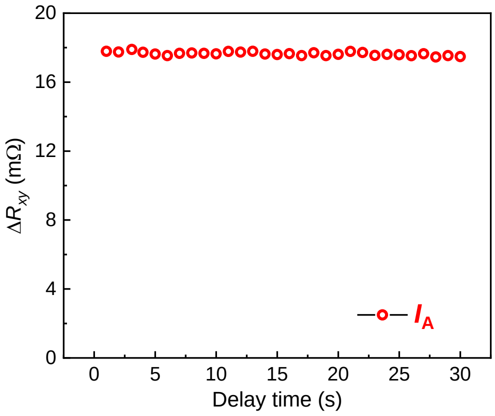

Supplementary Fig. 10: Waiting time measurement. After applying a 1-ms writing current density J_e = 1.5 × 10^7 A cm^{-2}, the planar Hall resistance ΔR_{xy} is measured every 1 s from 1 s to 30 s.

Supplementary Note 12: Thermal effect for SOT switching.

The thermal effect plays an important role in the spin-orbit torque (SOT) switching. In our work, the SOT switching current density is about 2 times of the current density for inducing the AFM-FM phase transition by Joule heating [$T_{\mathrm{AFM\text{-}FM}}$ = 300 K(base temperature) + 70 K(heating effect) = 370 K], which contributes to a 4 times heating power/thermal energy, and we can estimated the device temperature T_{SOT} during the SOT switching is around 580 K [T_{SOT} = 300 K(base temperature) + 4 × 70 K(heating effect)], as a result, the strong thermal disturbance will reduce the coercive field H_c from 300 Oe at 400 K to 27.3 Oe at 580 K, as shown in the *M-H* curves measured at different temperatures in Supplementary Fig. 11, so that the SOT effective field (48 Oe, for Ta: 34.4 Oe per 10^7 A cm^{-2})[7] can switch the magnetic order by the thermal disturbance.

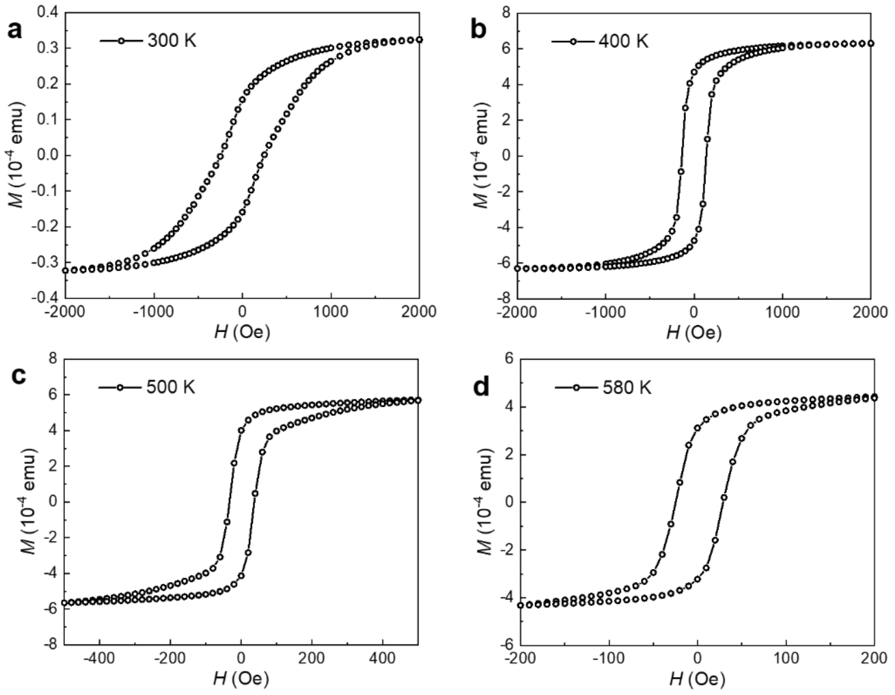

Supplementary Fig. 11: Thermal effect during SOT switching. *M-H* curves for the FeRh(20)/Ta/Ir sample measured at varied temperatures of 300 K (**a**), 400 K (**b**), 500 K (**c**), 580 K (**d**).

Supplementary Note 13: Energy dispersive spectroscopy.

We have measured the energy dispersive spectroscopy (EDS) from the area #1 of the cross-section TEM data in the MgO//FeRh/Ta/Ir sample, as shown in Supplementary Fig. 12. From the EDS spectrum, we can obtain the atomic ratio Fe : Rh= 1.03 : 1.

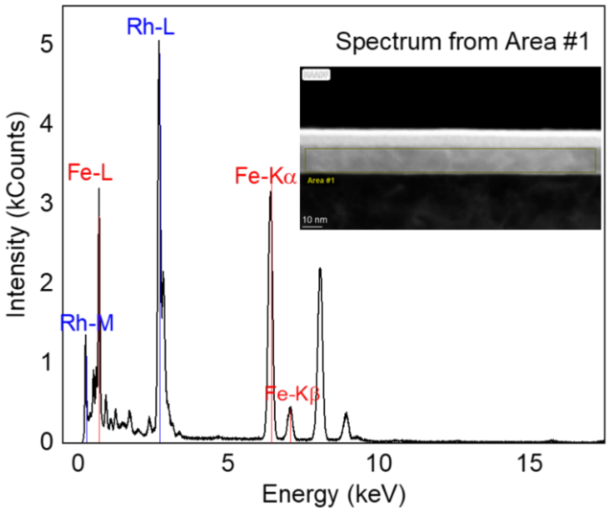

Supplementary Fig. 12: Energy dispersive spectroscopy. Energy dispersive spectroscopy (EDS) from the area #1 of the cross-section TEM data in the MgO//FeRh/Ta/Ir sample.

Supplementary Note 14: Polarity dependences of the writing current.

Actually, for the 90° Néel vector switching, positive and negative currents (180° reversal) give the similar switching behavior, because of the preserved 180° inversion symmetry of the Néel vector. As shown in Supplementary Fig. 13, for the opposite (negative) writing current direction (along the -*y* and the -*x* directions, respectively) with Fig. 2 (along the +*y* and the +*x* directions, respectively) in the manuscript, the switching polarity of the planar Hall resistance (Néel vector) is the same.

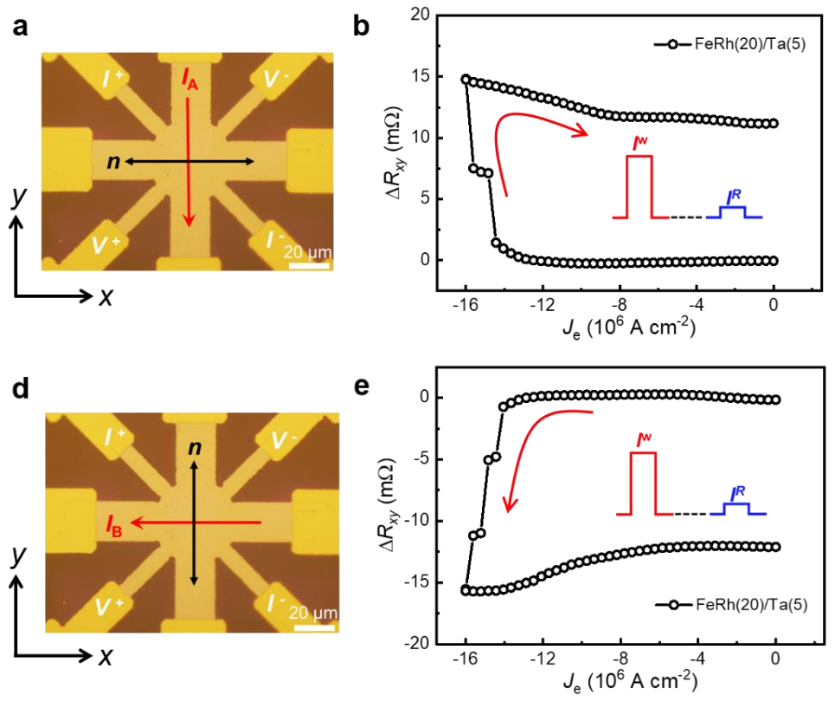

Supplementary Fig. 13: Polarity dependences of the writing current. Current-driven planar Hall resistance (Néel vector) switching for the writing current along the -*y* (**a**) and the -*x* (**b**) directions, respectively.

Supplementary Note 15: Oersted field contribution.

Supplementary Fig. 14 shows the Oersted field distribution in the cross-section region of the film stack FeRh(20)/Ta(5)/Ir(2), for the $J_e = 1.4 \times 10^7$ A cm^{-2}, with an average Oersted field value of 1.4 Oe for FeRh by the integration inside the FeRh region, which is more than 30 times smaller than the SOT effective field of 48 Oe (for Ta: 34.4 Oe per 10^7 A cm^{-2})[7]. Therefore, SOT contributes to the Néel vector switching, not the Oersted field.

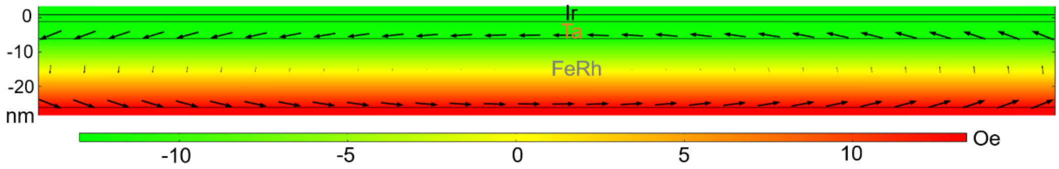

Supplementary Fig. 14: Oersted field distribution. The field distribution in the cross-section region of the film stack FeRh(20)/Ta(5)/Ir(2), for the $J_e = 1.4 \times 10^7$ A cm^{-2}.

References:

1 Wang, H. L. *et al.* Scaling of Spin Hall Angle in 3d, 4d, and 5d Metals from $Y_3Fe_5O_{12}$/Metal Spin Pumping. *Physical Review Letters* **112**, 197201 (2014).

2 Baldasseroni, C. *et al.* Temperature-driven growth of antiferromagnetic domains in thin-film FeRh. *Journal of Physics: Condensed Matter* **27**, 256001 (2015).

3 Scholl, A. *et al.* Observation of Antiferromagnetic Domains in Epitaxial Thin Films. *Science* **287**, 1014-1016 (2000).

4 Nolting, F. *et al.* Direct observation of the alignment of ferromagnetic spins by antiferromagnetic spins. *Nature* **405**, 767-769 (2000).

5 Kuiper, P., Searle, B. G., Rudolf, P., Tjeng, L. H. & Chen, C. T. X-ray magnetic dichroism of antiferromagnet Fe_2O_3: The orientation of magnetic moments observed by Fe 2p x-ray absorption spectroscopy. *Physical Review Letters* **70**, 1549-1552 (1993).

6 Grzybowski, M. J. *et al.* Imaging Current-Induced Switching of Antiferromagnetic Domains in CuMnAs. *Physical Review Letters* **118**, 057701 (2017).

7 Wu, H. *et al.* Room-Temperature Spin-Orbit Torque from Topological Surface States. *Physical Review Letters* **123**, 207205 (2019).
